# Machine learning algorithms for activity recognition in ambulant children and adolescents with cerebral palsy

**DOI:** 10.1186/s12984-018-0456-x

**Published:** 2018-11-15

**Authors:** Matthew Ahmadi, Margaret O’Neil, Maria Fragala-Pinkham, Nancy Lennon, Stewart Trost

**Affiliations:** 1 Institute of Health and Biomedical Innovation at QLD Centre for Children’s Health Research, School of Exercise and Nutrition Sciences, Queensland University of Technology, 62 Graham St, South Brisbane, QLD 4101 Australia; 2 Department of Physical Therapy and Rehabilitation Sciences, Drexel University, 1601 Cherry St., Philadelphia, PA USA; 3 Research Center, Franciscan Children’s Hospital, 30 Warren St., Brighton, MA USA; 4 Gait Analysis Laboratory, AI DuPont Hospital for Children, 1600 Rockland Rd, Wilmington, DE USA

**Keywords:** Accelerometers, Wearable sensors, Placement, Feature fusion, Habitual physical activity, GMFCS level, CP

## Abstract

**Background:**

Cerebral palsy (CP) is the most common physical disability among children (2.5 to 3.6 cases per 1000 live births). Inadequate physical activity (PA) is a major problem effecting the health and well-being of children with CP. Practical, yet accurate measures of PA are needed to evaluate the effectiveness of surgical and therapy-based interventions to increase PA. Accelerometer-based motion sensors have become the standard for objectively measuring PA in children and adolescents; however, current methods for estimating physical activity intensity in children with CP are associated with significant error and may dramatically underestimate HPA in children with more severe mobility limitations. Machine learning (ML) models that first classify the PA type and then predict PA intensity or energy expenditure using activity specific regression equations may be more accurate than standalone regression models. However, the feasibility and validity of ML methods has not been explored in youth with CP. Therefore, the purpose of this study was to develop and test ML models for the automatic identification of PA type in ambulant children with CP.

**Methods:**

Twenty two children and adolescents (mean age: 12.8 ± 2.9 y) with CP classified at GMFCS Levels I to III completed 7 activity trials while wearing an ActiGraph GT3X+ accelerometer on the hip and wrist. Trials were categorised as sedentary (SED), standing utilitarian movements (SUM), comfortable walking (CW), and brisk walking (BW). Random forest (RF), support vector machine (SVM), and binary decision tree (BDT) classifiers were trained with features extracted from the vector magnitude (VM) of the raw acceleration signal using 10 s non-overlapping windows. Performance was evaluated using leave-one-subject out cross validation.

**Results:**

SVM (82.0–89.0%) and RF (82.6–88.8%) provided significantly better classification accuracy than BDT (76.1–86.2%). Hip (82.7–85.5%) and wrist (76.1–82.6%) classifiers exhibited comparable prediction accuracy, while the combined hip and wrist (86.2–89.0%) classifiers achieved the best overall performance. For all classifiers, recognition accuracy was excellent for SED (94.1–97.9%), good to excellent for SUM (74.0–96.6%) and brisk walking (71.5–86.0%), and modest for comfortable walking (47.6–70.4%). When comfortable and brisk walking were combined into a single walking class, recognition accuracy ranged from 90.3 to 96.5%.

**Conclusions:**

ML methods provided acceptable classification accuracy for detection of a range of activities commonly performed by ambulatory children with CP. The resultant models can help clinicians more effectively monitor bouts of brisk walking in the community. The results indicate that 2-step models that first classify PA type and then predict energy expenditure using activity specific regression equations are worthy of exploration in this patient group.

**Electronic supplementary material:**

The online version of this article (10.1186/s12984-018-0456-x) contains supplementary material, which is available to authorized users.

## Background

Cerebral palsy (CP) is the most common physical disability of childhood with a prevalence of 2.5 to 3.6 cases per 1000 live births [1]. Inadequate physical activity is a major problem impacting the health status, functional mobility and well-being of children with CP [2, 3]. Moreover, low levels of physical activity may contribute to the development of disabling secondary conditions such as obesity, chronic pain, fatigue, and osteoporosis [4, 5]. In light of this evidence, strategies and goals for rehabilitation services have shifted from a focus on developmental motor skills to interventions to improve functional mobility and habitual physical activity (PA) [2, 3, 6]. To evaluate the effectiveness of such interventions, researchers and clinicians have relied on self-reports of PA. Although self-reports are low-cost and convenient to complete, they are subject to considerable social desirability and recall bias, and therefore may not be sufficiently valid or reliable for assessment of clinically important changes in PA [7].

Given the limitations of self-reports, accelerometer-based motion sensors have become the method of choice for assessing PA in children and adolescents [8]. However, despite their widespread use among children with typical development, calibrating accelerometer output to units of energy expenditure or physical activity intensity in youth with CP presents significant methodological challenges. The motor impairments and decreased mechanical efficiency of children with CP mandates that algorithms to delineate physical activity type or intensity from accelerometer output be specifically developed for the CP population [9].

To date, a number of investigators have completed studies deriving CP-specific intensity-based thresholds or cut-points for categorising processed accelerometer output (activity counts) as sedentary (SED), light (LPA), or moderate-to-vigorous physical activity (MVPA) [10–14]. While cut-point methods have enabled researchers and clinicians to objectively measure and describe HPA levels in youth with CP, recent work suggests that existing cut-points for children and adolescents with CP are associated with significant misclassification error and may dramatically underestimate the HPA levels of children with more severe mobility limitations [14]. Accordingly, there is a critical need to explore new accelerometer data processing methods that have the potential to provide more accurate assessments of HPA in youth with CP.

Pattern recognition methodologies, such as those utilising machine learning approaches, have the potential to significantly improve the accuracy of accelerometer-based assessments of PA. Studies involving children with typical development have shown that standard machine learning algorithms provide accurate predictions of activity type and intensity [15–18]. Importantly, the energy expenditure prediction errors associated with these models are 25 to 50% smaller in magnitude than those obtained with regression-based cut-point methods [16]. Two-step models that first classify activity type and then predict energy expenditure using activity specific regression equations may be more accurate than standalone regression models [19–21]. However, to our knowledge, no previous study has evaluated the feasibility and validity of machine learning methods to predict physical activity type in children with CP.

To address this gap in knowledge, the purpose of this study was to develop and test machine learning models to predict physical activity type in ambulant children and adolescents with CP. Three state-of-the-art supervised learning algorithms were evaluated – binary decision trees, support vector machines, and random forests. If physical activity type can be detected with acceptable accuracy in this patient group, then 2-step models employing activity-specific energy expenditure prediction equations may be a viable alternative to conventional cut-point methods. To determine the effects of accelerometer placement on recognition accuracy, we compared models trained on accelerometer data collected on the hip, wrist, and the combination of the hip and wrist.

## Methods

### Participants

Participants were recruited from the outpatient CP clinics at Franciscan Children’s Hospital, and Nemours AI duPont Hospital for Children. The inclusion criteria were as follows: diagnosis of CP at Gross Motor Function Classification System (GMFCS) level I, II, or III; between the ages of 6–20 years; and able to follow directions. Parents and/or health care providers (doctors or therapists) verified that participants were able to follow directions and complete the study protocols. Participants were excluded from the study if they had: undergone orthopaedic surgery within the last 6 months of the study start date; received lower extremity botulinum toxin injections within 3 months of the study start date; or experienced a recent musculoskeletal injury or had a medical condition limiting their ability to complete the physical activity protocol. A total of 22 ambulatory youth with CP participated in the study. Descriptive characteristics are displayed in Table 1. The study was approved by the university’s institutional review board (1111000396). Prior to participation, parents provided written consent and children written assent.

**Table 1:** Participants Characteristics

	Boys	Girls	Total
N	11	11	22
Age (years)	13.1 ± 3.3	12.6 ± 2.8	12.9 ± 3.0
Height (cm)	151.2 ± 16.1	145.7 ± 14.4	148.5 ± 15.2
Weight (kg)	45.2 ± 12.8	40.8 ± 12.6	43.0 ± 12.6
GMFCS:			
I	5	8	13
II	6	1	7
III	0	2	2

### Data collection

Participants completed seven standardized activity trials while wearing an ActiGraph GT3X+ tri-axial accelerometer (ActiGraph Corporation, Pensacola, FL) on the hip and wrist that they used most in activities of daily living [14]. A complete description of each trial and the data collection protocols have been published previously [22, 23]. The activity trials were completed in a single two-hour session and comprised the following sequence of activities: 1) Supine rest (lying down resting but not sleeping); 2) Seated handwriting (sitting in a chair at a desk, using a pencil or pen to transcribe a standardized written script to a pad of paper); 3) Wiping down a countertop (walking from side to side in front of a 2 m long countertop spraying and wiping to clean the entire surface); 4) Folding laundry (loading a laundry bag with five towels and carry it 3 m; dumping out the towels, folding them, loading them back in the bag, carrying it back to the original starting spot, and repeating); 5) Comfortable walk (walking at a comfortable self-selected speed after receiving the instructions “walk at a comfortable pace like when you are at the mall or walking in your neighbourhood or at school but not in a hurry”); 6) Brisk walk (walking at a brisk speed after receiving the instructions “this time we want you to walk a little faster so please walk at a faster pace like when you are hurrying to get to class after the bell has rung”); 7) Fast walk (walking at a fast speed after receiving the instructions “this time we want you to walk as fast as you possibly can without falling or running”). All walking trials were completed on a 25 m course marked with two cones. Prior to each walking trial, participants completed a brief practice test to help them select an appropriate walking speed. Additionally, during each walking trial, a physical therapist walked alongside each participant to help them maintain a consistent pace throughout the walking trial. Activity trials 1 to 4 were 5 min in duration, while trials 5 to 7 were 6 min in duration. For the purpose of developing the physical activity classifiers, the activity trials were categorized into four activity classes: sedentary (SED) (supine rest, seated handwriting); standing utilitarian movements (SUM) (wiping down countertop, folding laundry); comfortable walking (CW) (comfortable walk); and brisk walking (BW) (brisk walk and fast walk). All data collected during the wiping down the countertop trials and laundry trial were annotated as SUM activities even though they included brief episodes of walking.

### Instrumentation

The ActiGraph GT3X+ measures acceleration along three perpendicular axes ranging in magnitude from +/− 6 G. The accelerometer output is sampled at a user-specified rate between 30 and 100 Hz and stored in non-volatile flash memory for downloading and post processing. For the current study, a sampling frequency of 30 Hz was used. The acceleration signal from each axis was transformed into a single dimension vector magnitude (VM) using the equation:

$$ VM=\surd \left({x}^2+{y}^2+{z}^2\right) $$

### Data pre-processing and feature extraction

Standard visual data screening tools (e.g., histograms, boxplots) were used to identify missing data or potential outliers. No missing or abnormal values were found. Features from the VM were extracted from 10 s non-overlapping windows. We extracted features from non-overlapping windows of 10 s duration to replicate classifiers developed in typically developing children [16, 24]; and to provide sufficient time resolution to detect the intermittent activity patterns of children. A total of 27 time and frequency domain features shown to be beneficial in previous activity recognition studies were extracted [24, 25]. The selected time domain features were as follows: minimum, maximum, sum, power, log energy, peak to peak, lag one autocorrelation, mean, standard deviation, coefficient of variation, median crossings, percentiles (10th,25th,50th,75th,90th), and interquartile range. The selected frequency domain features included signal entropy between 0.25 and 5.0 Hz and the top 3 dominant frequencies, magnitudes, and frequency power ratios (the percentage of the total signal power accounted for by the dominant frequency) between 0.25 and 5.0 Hz.

### Machine learning algorithms

Three supervised learning algorithms were used to build the activity classifiers: Binary Decision Tree (BDT), Random Forest (RF), and Support Vector Machine (SVM). Each algorithm is briefly described below. A more detailed description of these algorithms can be found elsewhere [26–28].

BDT’s use a recursive partitioning technique for classification. The tree begins with a single root node and splits into branches, leading to further nodes until a leaf node is reached. Every non-leaf node is associated with a binary decision which determines which branch to follow. The decision tree is built by identifying a feature value that split the training data into subgroups with the greatest class purity. The splitting continues in each node until the class subgroups reach a minimum size or until a stop condition is reached. In the current study, splits were based on the information gain index which identifies the split point providing the greatest reduction in entropy or label impurity. The complexity parameter, which prevents overfitting by regulating the depth of the tree, was optimised during cross-validation. BDT was selected because it is relatively easy to implement and the results can be translated into rules that can be applied by most researchers and clinicians.

RF is an ensemble of multiple decision trees. Each tree is learned on a bootstrap sample of training data and each node in the tree is split using the best among a randomly selected set of features. The decisions from each tree are aggregated and a final model prediction is based on majority vote [27]. The RF model requires very little pre-processing of the data, as the features do not need to be normalized and feature selection procedures are not required because the algorithm effectively does this on its own. Additionally, the model is resistant to over fitting the training data because each tree within the forest is independently grown to maximum depth using a randomly selected subset of features.

SVM’s perform classification tasks by mapping features onto a multidimensional space and constructing decision boundaries, called hyperplanes, which maximise the margin between observations of different activity classes. The margin is determined by the distance between “support vectors”, which are observations that lie in an area of space which creates a boundary between activity classes. New observations are mapped onto the multidimensional space and assigned a class prediction based on which side of the hyperplane it lies [26]. We chose to use an SVM model because it performs well with high dimensional data, is robust to noise, and it is able to classify non-linearly separable feature vectors using Kernel functions. The SVM classifiers were configured using a radial basis kernel function, and the cost parameter was optimized in the course of cross-validation. The cost parameter or soft margin adjusts the width of the hyperplane. For implementation of the SVM models, features were normalized to a mean of zero and scaled to unit variance by subtracting the corresponding mean and dividing by the standard deviation.

### Model training and cross-validation

Models were trained and cross-validated using the “kernlab”, “randomForest”, “rpart”, and “caret” packages within R (Version 3.3.2) [29]. Model fit was evaluated using leave-one-subject-out (LOSO) cross-validation. With LOSO cross validation, a model is trained on data from all participants except one, who is “left out” and used as a test dataset. The process is repeated until each participant has been used as a test dataset, and the results are aggregated. The cross-validation performances of the classifiers were evaluated by computing overall accuracy and F-scores.

Overall accuracy was calculated as:

$$ \frac{correctly\ classified\ observations}{total\ observations}\times 100 $$

F-score was calculated as:

$$ \frac{\left(2\times Precision\times Recall\right)}{\left( Precision+ Recall\right)}\times 100 $$

Recall measures the proportion of observations that were correctly classified (equivalent to sensitivity), while precision measures the proportion of predicted observations that were correct (equivalent to positive predictive value). F-scores were calculated because it is based on the harmonic mean of precision and recall and is less biased by class size imbalances [30]. F-scores were calculated for each activity class and averaged to provide an overall F-score for each classifier. Additionally, for each model, confusion matrices were generated to summarise patterns of misclassification for each classifier.

### Statistical analysis

Differences in overall accuracy and F-scores were tested for statistical significance using Friedman nonparametric tests as described by Stopor [31]. When the test statistic was significant, pair-wise tests with a Bonferroni correction for multiple comparisons was used to determine the location of significant differences. An alpha level of 0.05 was used as the level of significance.

## Results

In the BDT models, the complexity parameter was optimized at 0.01. The complete BDT models for each placement configuration are displayed in Additional file 1. The RF models were built with 500 trees and the number of features randomly sampled at each split was optimized at 3 for the wrist and hip models and 6 for the combined hip and wrist model. For the SVM models, the cost parameter was optimized at 3.0.

**Fig. 1:**
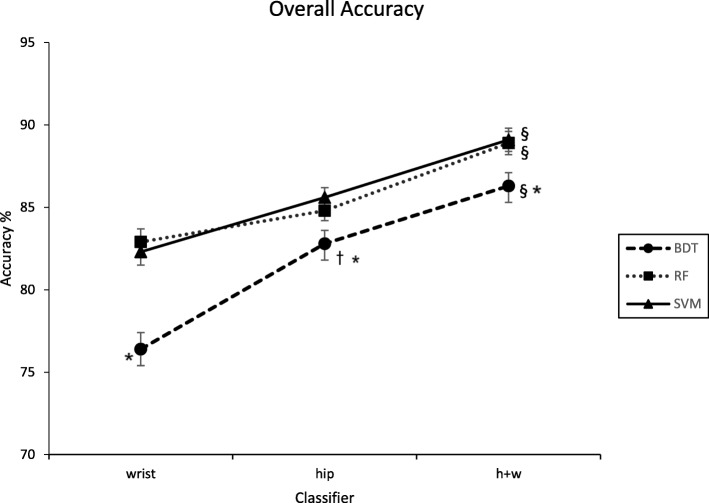
Overall accuracy performance for hip, wrist, and combined hip and wrist classifiers. BDT = Binary Decision Tree, RF = Random Forest, SVM = Support Vector Machine, h+w = hip and wrist, * = significantly different from RF and SVM at a given placement location, † = significantly different from wrist for a given algorithm, § = significantly different from hip or wrist for a given algorithm

Classification accuracy for each supervised learning algorithm and accelerometer configuration are displayed in Fig. 1. The Friedman nonparametric test provided statistical evidence of a significant difference in classification accuracy across the nine models (F_8,168_ = 14.1, *p* < .0001). For all three placement configurations, SVM and RF exhibited significantly better classification accuracy than BDT. For BDT, the combined hip and wrist classifier exhibited significantly higher classification accuracy than the hip classifier, with the hip classifier, in turn, providing significantly higher classification than the wrist. For SVM and RF, the combined hip and wrist classifiers exhibited significantly higher classification accuracy than the hip or wrist classifiers. Although SVM and RF classifiers trained on hip data exhibited higher classification accuracy than those trained on wrist data, the differences in overall accuracy were small (1.9–3.3%) and not statistically significant.

**Fig. 2:**
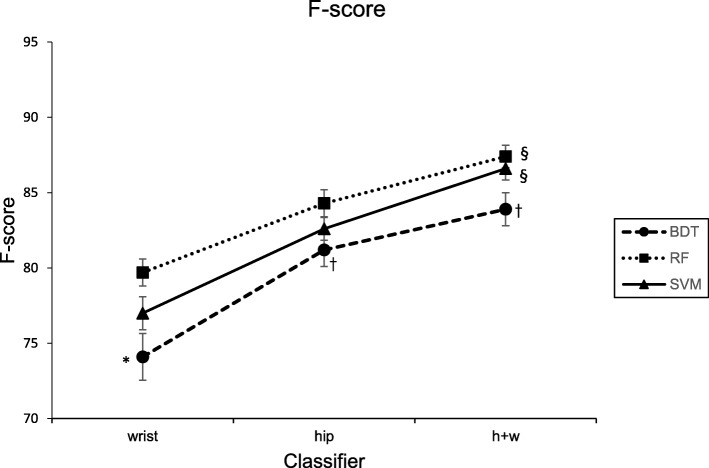
F-score performance for hip, wrist, and combined hip and wrist classifiers. BDT = Binary Decision Tree, RF = Random Forest, SVM = Support Vector Machine, h+w = hip and wrist, * = significantly different from RF at a given placement location, † = significantly different from wrist for a given algorithm, § = significantly different from hip or wrist for a given algorithm

Average F-scores for each supervised learning algorithm and accelerometer configuration are displayed in Fig. 2. The Friedman nonparametric test provided statistical evidence of a significant difference in average F-scores across the nine models (F_8,168_ = 11.5, p < .0001). At the wrist location, RF exhibited significantly better performance than BDT. However, for the hip and combined hip and wrist, average F-scores for BDT, SVM and RF classifiers were not statistically significant. For BDT, the hip and combined hip and wrist classifiers exhibited significantly better performance than the wrist classifier. For SVM and RF, the combined hip and wrist classifiers exhibited significantly better performance than the hip or wrist classifiers. SVM and RF models trained on hip data exhibited higher average F-scores than those trained on wrist data; however, the performance differential was not significantly different.

Confusion matrices for the RF, SVM, and BDT classifiers are presented in Tables 2, 3 and 4, respectively. Recognition accuracy for SED activities was excellent (94.1–97.9%), good to excellent for SUM (74.0–96.6%) and BW (71.5–86.0%), but modest for CW (47.6–70.4%), particularly for the wrist classifiers. The combined hip and wrist models provided consistently higher recognition accuracy for each activity class than the individual hip and wrist classifiers. For all three learning algorithms, a significant percentage of comfortable paced walking instances (21.1–39.9%) were misclassified as fast paced walking, with the misclassification rate being highest for classifiers trained on wrist data. However, when the comfortable- and brisk-paced walking classes were combined into a single walking class, the average recognition accuracy for walking ranged from 90.3 to 96.5%.

**Table 2:** Confusion matrices for Binary Decision Tree, Random Forest, and Support Vector Machine classifiers trained on wrist data

Activity Class	Binary Decision Tree			
	Observed			
Prediction	SED	SUM	CW	BW
1. SED	**1294 [0.95]**	70 [0.05]	0 [0.00]	0 [0.00]
2. SUM	47 [0.03]	**1009 [0.74]**	124 [0.09]	184 [0.13]
3. CW	7 [0.01]	90 [0.12]	**370 [0.48]**	310 [0.40]
4. BW	18 [0.01]	197 [0.13]	163 [0.10]	**1176 [0.76]**
Random Forest				
1. SED	**1311 [0.96]**	47 [0.03]	0 [0.00]	6 [0.00]
2. SUM	30 [0.02]	**1267 [0.93]**	16 [0.01]	51 [0.04]
3. CW	7 [0.01]	75 [0.10]	**442 [0.57]**	253 [0.33]
4. BW	20 [0.01]	135 [0.09]	242 [0.16]	**1157 [0.74]**
Support Vector Machine				
1. SED	**1327 [0.97]**	37 [0.03]	0 [0.00]	0 [0.00]
2. SUM	51 [0.04]	**1242 [0.91]**	28 [0.02]	43 [0.03]
3. CW	9 [0.01]	112 [0.14]	**467 [0.60]**	189 [0.24]
4. BW	17 [0.01]	170 [0.11]	256 [0.16]	**1111 [0.71]**

Numbers represent observation counts. Percentage of observations for a given class reported in brackets. Values in bold face indicate number and proportion of observations within each class correctly classified

*SED* sedentary, *SUM* standing utilitarian movements, *CW* comfortable walk, *BW* brisk walk

**Table 3:** Confusion matrices for Binary Decision Tree, Random Forest, and Support Vector Machine classifiers trained on hip data

Activity Class	Binary Decision Tree			
	Observed			
Prediction	SED	SUM	CW	BW
1. SED	**1294 [0.95]**	70 [0.05]	0 [0.00]	0 [0.00]
2. SUM	149 [0.11]	**1138 [0.83]**	72 [0.05]	5 [0.00]
3. CW	7 [0.01]	57 [0.07]	**534 [0.69]**	179 [0.23]
4. BW	17 [0.01]	66 [0.04]	255 [0.16]	**1216 [0.78]**
Random Forest				
1. SED	**1304 [0.96]**	59 [0.04]	1 [0.00]	0 [0.00]
2. SUM	127 [0.09]	**1193 [0.87]**	26 [0.02]	18 [0.01]
3. CW	7 [0.01]	65 [0.08]	**471 [0.61]**	234 [0.30]
4. BW	16 [0.01]	51 [0.03]	168 [0.11]	**1319 [0.85]**
Support Vector Machine				
1. SED	**1310 [0.96]**	51 [0.04]	1 [0.00]	2 [0.00]
2. SUM	149 [0.11]	**1138 [0.83]**	72 [0.05]	5 [0.00]
3. CW	7 [0.01]	57 [0.07]	**534 [0.69]**	179 [0.23]
4. BW	18 [0.01]	47 [0.03]	188 [0.12]	**1301 [0.84]**

Numbers represent observation counts. Percentage of observations for a given class reported in brackets. Values in bold face indicate number and proportion of observations within each class correctly classified

*SED* sedentary, *SUM* standing utilitarian movements, *CW* comfortable walk, *BW* brisk walk

**Table 4:** Confusion matrices for Binary Decision Tree, Random Forest, and Support Vector Machine classifiers trained on combined hip and wrist data

Activity Class	Binary Decision Tree			
	Observed			
Prediction	SED	SUM	CW	BW
1. SED	**1284 [0.94]**	80 [0.06]	0 [0.00]	0 [0.00]
2. SUM	45 [0.03]	**1243 [0.91]**	70 [0.05]	6 [0.00]
3. CW	4 [0.01]	62 [0.08]	**547 [0.70]**	164 [0.21]
4. BW	10 [0.01]	74 [0.05]	185 [0.12]	**1285 [0.83]**
Random Forest				
1. SED	**1326 [0.97]**	36 [0.03]	0 [0.00]	2 [0.00]
2. SUM	20 [0.01]	**1317 [0.97]**	11 [0.01]	16 [0.01]
3. CW	4 [0.01]	52 [0.07]	**511 [0.66]**	210 [0.27]
4. BW	10 [0.01]	43 [0.03]	164 [0.11]	**1337 [0.86]**
Support Vector Machine				
1. SED	**1335 [0.98]**	27 [0.02]	1 [0.00]	1 [0.00]
2. SUM	34 [0.02]	**1316 [0.96]**	7 [0.01]	7 [0.01]
3. CW	4 [0.01]	71 [0.09]	**529 [0.68]**	173 [0.22]
4. BW	7 [0.00]	50 [0.03]	176 [0.11]	**1321 [0.85]**

Numbers represent observation counts. Percentage of observations for a given class reported in brackets. Values in bold face indicate number and proportion of observations within each class correctly classified

*SED* sedentary, *SUM* standing utilitarian movements, *CW* comfortable walk, *BW* brisk walk

## Discussion

To our knowledge, this is the first study to develop and test machine learning models for the automatic identification of physical activity type in ambulant children with CP. Our classifiers trained on features from the VM of the raw acceleration signal from the hip, wrist, and combination of the hip and wrist achieved acceptable recognition accuracy for a range of physical activities that are routinely performed by ambulant children with CP. Notably, our classifiers were able to accurately detect bouts of brisk walking, which is an important motor activity to promote function and participation, and a predictor of clinically important changes in quality of life [2, 32]. Further, our classifiers developed for children with CP displayed comparable performance accuracy to those trained in typically developing children [17, 24, 33–35]. These findings support the feasibility and utility of machine learning approaches to accelerometer data processing in children with CP and indicates that 2-step models that first classify the activity type and then predict energy expenditure using activity specific regression equations are worthy of future exploration in this patient group.

The RF and SVM classifiers exhibited significantly overall higher classification accuracy than the BDT classifiers. This finding is consistent with the results of a study involving typically developing children in which RF and SVM classifiers outperformed BDT in relation to recognition of seven commonly performed child activities [33]. Although intuitive and easy to implement, decision trees generally exhibit lower classification accuracy than more sophisticated machine learning algorithms [36]. This is because the strictly horizontal and vertical decision boundaries created by decision trees lack precision, resulting in variable predictions and increased error when applied to new data. RF models endeavour to overcome this limitation by building large numbers of decision trees using different sets of bootstrapped training data, randomly selecting subsets of features, and basing final class predictions on majority vote. SVM models provide more precise decision boundaries through the use of regularisation parameters which allow “soft margins” and specialised kernel functions which transform features into a higher dimensional space. This enables SVM models to linearly separate data points that would otherwise not be linearly separable, providing superior performance in noisy and high-dimensional data. Although RF and SVM models outperformed BDT in the current study, it is important to consider the “no free lunch theorem” which states that there is no one model that works best for every problem [37]. Consequently, future studies should evaluate the utility of other supervised learning algorithms or consider implementing a customised ensemble in which the decisions of multiple classifiers are fused [33, 38].

The results demonstrate that activity classifiers trained on data from a single accelerometer worn on the hip or wrist can accurately detect activity type in ambulatory children with CP. Although accuracy for the hip classifiers were, on average, 3.9 percentage points higher than the wrist classifiers, performance differences between the hip and wrist were not statistically significant (with the exception of BDT which exhibited the lowest performance of the three supervised learning algorithms). The performance differential for the hip and wrist classifiers is similar to that observed in a previous investigation comparing hip and wrist classifiers in typically developing children [24]. In that study, regularized logistic regression classifiers trained on hip and wrist accelerometer data displayed a difference in recognition accuracy of just under 3%, although the number of target classes was higher. The practical significance of the small performance differential in favour of the hip is not clear, particularly if the activities with favourable detection accuracy at the hip location account for a relatively small portion of the monitoring day [39].

Consistent with the results of studies conducted in adults [40] and typically developing children [17], the combined hip and wrist classifiers provided consistently higher recognition accuracy than the single sensor classifiers. Inspection of the confusion matrices revealed that the wrist classifiers were superior at detecting activities involving significant arm movement (e.g. wiping down a countertop), while the hip classifiers were better at detecting locomotor activities. Therefore, by mitigating the weaknesses one sensor location may have for detection of certain activities, the fusion of features from multiple sensor locations resulted in improved activity recognition across a range of activity types.

In the current study, the activity recognition models exhibited high recognition accuracy for brisk walking (72–86%). This is an important finding with significant implications for clinical practice because the majority of youth with CP have difficulty walking due to associated impairments such as spasticity, weakness, and decreased postural control [41]. Goals of therapy are to improve activity limitations (i.e., decreased walking speed and endurance), increase gait speed, and promote function and greater participation [41, 42]. Physical therapy interventions such as exercise, treadmill and overground ambulation training are often used to improve gait speed and endurance [41, 42]. Therefore, automatic recognition of brisk walking in free-living contexts is critical in monitoring the effectiveness of therapeutic interventions to promote efficient gait speed for increased HPA, function, and participation.

The present study had several limitations that should be acknowledged. First, the classifiers were evaluated under controlled activity trials which may not fully represent activity patterns in free-living conditions. Consequently, additional research is needed to evaluate the generalizability of these models in a free-living environment. Second, only two of the 22 participants were classified as GMFCS level III. The inclusion of more study participants with severe motor impairments would have strengthened the study design and increased the generalisability of the resultant classification models. Third, relative to brisk walking, recognition of comfortable paced walking was modest. This could be due to the limited set of features used to build the classifiers. It may be that other features from the acceleration signal not included in the current study could more accurately differentiate comfortable walking from brisk walking. Another reason for the low classification accuracy of comfortable walking could be that the SUM activities also included brief episodes of walking. Alternatively, the misclassification of comfortable walking and brisk walking may be related to our use of self-selected walking speed. Notably, the range of walking speeds achieved by children with more severe impairments (GMFCS II & III) during the brisk/fast walking trials (32.9–77.8 m/min) overlapped considerably with the comfortable walking speeds of children with less impairment (GMFCS I) (38.3–77.4 m/min). Consequently, future studies should investigate the prediction of other metrics related to locomotor performance such as walking speed, which can be used across all ambulatory GMFCS levels.

This study had a number of strengths. It is the first study to develop and evaluate machine learning activity recognition models for ambulatory youth with CP. Second, the study protocol comprised a mix of lifestyle and ambulatory activities commonly performed by children and adolescents. Third, a feature fusion method was implemented to develop a combined hip and wrist classifier that demonstrated higher classification accuracy than single hip and wrist models. Fourth, the study included participants with GMFCS levels I, II, and III, thus representing the full spectrum of ambulatory ability in CP.

## Conclusion

In summary, machine learning classifiers trained on accelerometer features from the wrist, hip and combined hip and wrist can be used to detect PA type in ambulant children and adolescents with CP. The RF and SVM classifiers consistently outperformed the BDT classifiers. The fusion of hip and wrist accelerometer features yielded significantly better performance, although single accelerometer classifiers trained on data from the hip or wrist provided acceptable classification accuracy. The results support the feasibility of machine learning approaches to accelerometer data processing in children with CP and the potential utility of 2-step models which first classify activity type and then predict energy expenditure using activity specific regression equations. Future studies should therefore: 1) develop classification models that, in addition to the activity classes examined in the current study, recognise activities performed by children with less severe motor impairments such as stair climbing and running; and 2) develop and test activity specific energy expenditure prediction models that account for the motor impairment and decreased mechanical efficiency of children with CP. If shown to be more accurate than single regression models or existing cut-point methods, the resultant models will enable researchers and clinicians to more effectively monitor the PA levels of their patients.

## Additional file

Additional file 1: AR.CP decision trees. (PDF 85 kb)

## Abbreviations

ANOVA: Analysis of variance
BDT: Binary decision tree
BW: Brisk walk
CP: Cerebral palsy
CW: Comfortable walk
GMFCS: Gross Motor Function Classification System
HPA: Habitual physical activity
LOSO: Leave-one-subject-out
LPA: Light physical activity
MVPA: Moderate to vigorous physical activity
RF: Random forest
SED: Sedentary behaviour
SUM: standing utilitarian movements
SVM: Support vector machine
VM: Vector magnitude
