# Supplementary material for: Machine learning algorithms for activity recognition in ambulant children and adolescents with cerebral palsy
Source: J Neuroeng Rehabil. 2018 Nov 15;15:105. doi: 10.1186/s12984-018-0456-x (PMC6238270; doi:10.1186/s12984-018-0456-x)
Supplement: Supplementary file 1 — AR.CP decision trees. (PDF 85 kb) [file 12984_2018_456_MOESM1_ESM.pdf]

Figure 1a

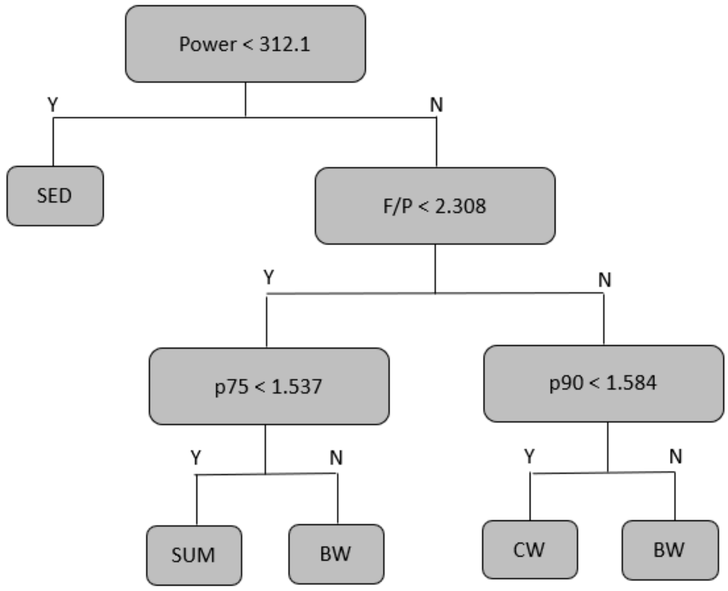

Power = sum of vector magnitude squared, F/P = Dominant Frequency Power Ratio of vector magnitude, p75 = 75th percentile, p90 = 90th percentile, SED = sedentary, SUM = standing utilitarian movements, CW = comfortable walk, BW = brisk walk

Figure 1b

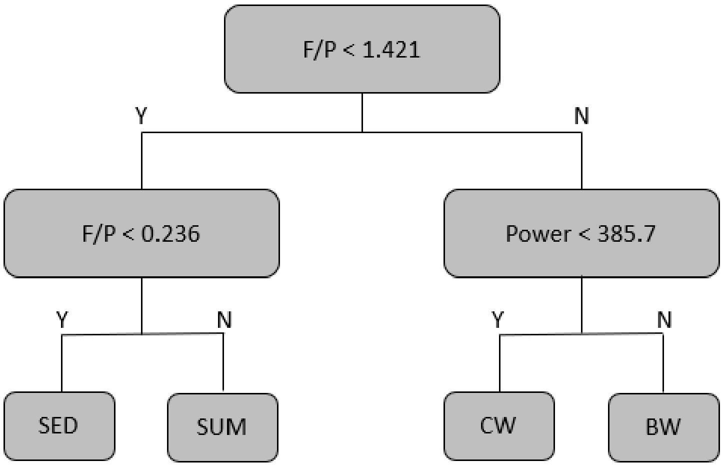

Power = sum of vector magnitude squared, F/P = Dominant Frequency Power Ratio of vector magnitude, SED = sedentary, SUM = standing utilitarian movements, CW = comfortable walk, BW = brisk walk

Figure 1c

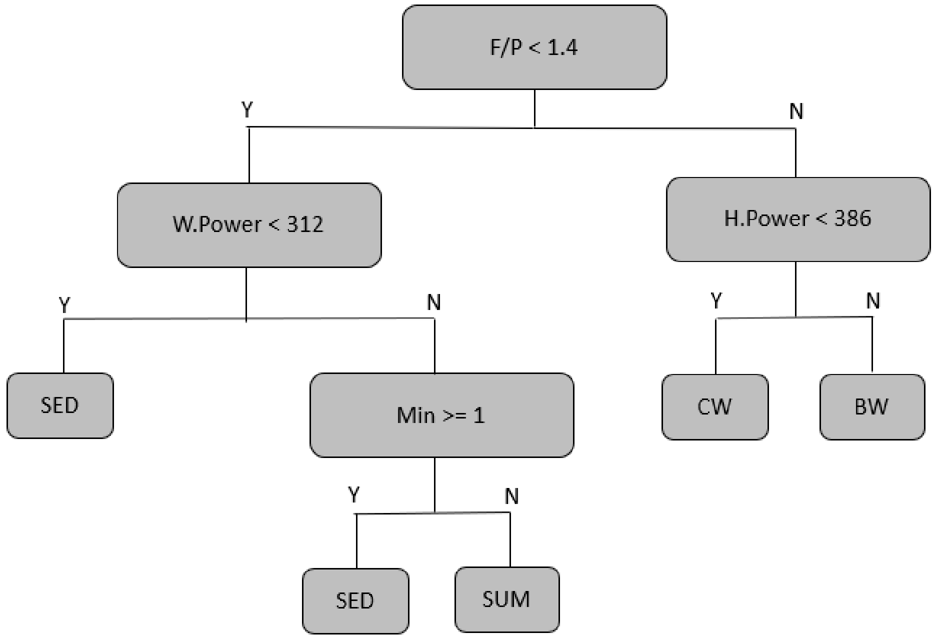

F/P = Dominant Frequency Power Ratio of vector magnitude, W.Power = sum of vector magnitude squared (wrist), H.Power = sum of vector magnitude squared (Hip), Min = minimum value of vector magnitude, SED = sedentary, SUM = standing utilitarian movements, CW = comfortable walk, BW = brisk walk
